# Complete genome sequence of *Paenibacillus polymyxa* 19197, an endophytic bacterium isolated from the morel mushroom *Morchella importuna*

**DOI:** 10.1128/mra.01171-25

**Published:** 2026-02-24

**Authors:** Changmeng Wu, Linsong He, Yingli Cai, Zhilan Xia, Fuqiang Yu, Xunyan He, Wei Liu

**Affiliations:** 1 College of Horticulture, Hunan Agricultural University 12575, Changsha, Hunan, China; 2 Institute of Subtropical Agriculture, Chinese Academy of Sciences 74541 , Changsha, Hunan, China; 3 School of Life Sciences, Yunnan University 674423 , Kunming, Yunnan, China; 4 Yunnan Key Laboratory for Fungal Diversity and Green Development & Yunnan International Joint Laboratory of Fungal Sustainable Utilization in South and Southeast Asia, Germplasm Bank of Wild Species, Kunming Institute of Botany, Chinese Academy of Sciences 26445 , Kunming, Yunnan, China; 5 Key Laboratory of Chemistry in Ethnic Medicinal Resources, School of Ethnic Medicine, Yunnan Minzu University 145309 , Kunming, Yunnan, China; 6 Huanjiang Agriculture Ecosystem Observation and Research Station of Guangxi, Guangxi Key Laboratory of Karst Ecological Processes and Services, Huanjiang Observation and Research Station for Karst Ecosystems, Chinese Academy of Sciences, Huanjiang, China; Wellesley College, Wellesley, Massachusetts, USA

**Keywords:** *Paenibacillus polymyxa*, endophyte, genome sequence

## Abstract

We report the complete genome sequence of *Paenibacillus polymyxa* 19197, an endophyte isolated from *Morchella importuna*. The 6.93 Mb circular chromosome encodes a diverse array of carbohydrate-active enzymes (CAZymes), including 220 glycoside hydrolases, and a complete nitrogenase operon, suggesting roles in polysaccharide degradation and nitrogen fixation within the morel ecosystem

## ANNOUNCEMENT

The commercial cultivation of the prized morel mushroom (*Morchella importuna*) faces yield instability, which is linked to microbial interactions in its growth environment (1–3). Endophytic bacteria, particularly *Paenibacillus* spp., are of interest for their metabolic versatility and plant growth-promoting properties (4). We isolated *Paenibacillus polymyxa* 19197 from *M. importuna* fruiting bodies. Its genome encodes a nitrogenase operon and polysaccharide-degrading enzyme genes, suggesting potential roles in nutrient cycling. To elucidate their genomic basis, we present the complete genome sequence of *P. polymyxa* 19197.

The strain was isolated from a surface-sterilized fruiting body (75% ethanol, 1.5 min) by plating internal tissue fragments on CYM agar. It was identified as *Paenibacillus polymyxa* based on 16S rRNA (5) gene sequencing using primers BSF (5′-GAGTTTGATCCTGGCTCAG-3′) and BSR (5′-AAGGAGGTGATCCAGCCGCA-3′), producing a 1,431 bp sequence (GenBank OR486028) with 99.65% identity to *Paenibacillus phytohabitans* (NR_181094), and confirmed by genomic analysis. Purification via repeated streaking minimized host DNA contamination, rendering additional removal steps unnecessary.

For genomic DNA extraction, a single colony of *P. polymyxa* 19197 was grown in liquid CYM medium at 23°C with shaking (200 rpm) to an OD₆₀₀ of 0.8. Cells were harvested by centrifugation (8,000× g, 10 min, 4°C), and the pellet was flash-frozen in liquid nitrogen and stored at −80°C. Genomic DNA was extracted using the cetyltrimethylammonium bromide (CTAB) method (6). The modifications included an additional incubation with proteinase K and a final purification step with RNase A treatment. DNA integrity was verified by 0.35% agarose gel electrophoresis, and concentration was measured using a Qubit Fluorometer.

For Illumina sequencing, a library was prepared with the TruSeq DNA Kit and sequenced on a NovaSeq platform, yielding 13,612,888 paired-end reads (2×151 bp; ~80× coverage). Reads were quality-controlled and adapter-trimmed using AdapterRemoval v2.2.2 (7). For Oxford Nanopore Technologies (ONT) sequencing, high-molecular-weight DNA (not mechanically sheared) was size-selected with BluePippin. A library was constructed with the SQK-LSK109 kit and sequenced on a PromethION device, yielding 277,223 raw reads (N50: 23.3 kb). Base calling was performed with Guppy v6.4.6 in high-accuracy mode.

Hybrid assembly was performed using Unicycler v0.4.8 (8) with Pilon v1.23 (9) polishing. The genome was submitted to DDBJ and annotated using DFAST (10). Carbohydrate-active enzymes were annotated using dbCAN2 (11), and secondary metabolite clusters with antiSMASH v6.0.0 (12).

The complete circular chromosome is 6,931,270 bp (GC content 51.0%). CheckM v1.2.1 (13) indicated 99.26% completeness and 0% contamination. The genome encodes 6,135 proteins, 86 tRNAs, and 27 rRNAs (Table 1); 92.2% of proteins were functionally annotated. Annotation identified: a complete nifHDK nitrogenase operon; chitinase (GH18, GH19) and cellulase (GH5, GH9) genes; 367 CAZymes (including 220 glycoside hydrolases) via dbCAN2 (11); and a lassopeptide cluster identical to the anti-MRSA paeninodin cluster via antiSMASH.

**TABLE 1:** Basic genomic characteristics of *Paenibacillus polymyxa* strain 19197

Genomic feature	Value
Accession Number	AP044781
BioProject	PRJDB37857
BioSample	SAMD01696271
Chromosome size (bp)	6,931,270
GC content (%)	51.0
Coding sequences (CDS)	6,135
tRNA genes	86
rRNA genes	27

## Data Availability

The complete genome sequence of *Paenibacillus polymyxa* 19197 has been deposited in DDBJ under accession number AP044781, which is publicly accessible at https://www.ddbj.nig.ac.jp/. The BioProject number is PRJDB37857. Raw sequencing data are available in the DDBJ Sequence Read Archive (DRA) under accession DRR801684.
